# Sexual recombination in cereal rust fungi: Knowns and unknowns of pathogen evolution and adaptation

**DOI:** 10.1371/journal.ppat.1012908

**Published:** 2025-02-04

**Authors:** Shideh Mojerlou, Benjamin Schwessinger, Julian Rodriguez-Algaba

**Affiliations:** 1 Department of Agroecology, Aarhus University, Slagelse, Denmark; 2 Research School of Biology, Australian National University, Canberra, Australia; Shanghai Center for Plant Stress Biology, CHINA

## 1. Why do sex and cereal rust fungi matter?

Cereal rust fungi, encompassing several species of *Puccinia*, pose a significant threat to global food security. Notable cereal rust pathogens on wheat include *P*. *graminis* f. sp. *tritici* (stem or black rust, *Pgt*), *P*. *striiformis* f. sp. *tritici* (stripe or yellow rust, *Pst*), and *P*. *triticina* (leaf or brown rust, *Pt*), while *P*. *hordei* (*Ph*) affects barley and *P*. *coronata* f. sp. *avenae* (*Pca*) infects oats [1]. The agricultural importance of rust fungi is deeply rooted in history, with archaeological evidence dating the presence of stem rust to over 3,300 years ago [2]. Ancient farmers sought divine intervention against rust fungi, with Romans establishing the deity Robigus to ward off rust-related disasters [3].

By the 17th century, farmers had recognized that severe cereal rust epidemics occurred near barberry shrubs (*Berberis* species). Despite barberry’s value as a medicinal plant, source of dyes, and popular hedging shrub, this observation led to the first barberry eradication laws in Rouen, France in 1660 [4]. In 1866, Anton de Bary proved that *P*. *graminis* required both wheat and barberry to complete its life cycle [5]. This discovery prompted widespread barberry eradication programs, including Denmark (1903) [6] and the United States of America (1918) [4]. However, the role of the sexual host in generating novel pathogen strains was not recognized until Craigie’s 1927 discovery of the function of the sexual structures in rust fungi [7]. Subsequent studies confirmed that rust strains with different patterns of infection on wheat varieties could arise through strain hybridization during sexual reproduction on the sexual host [8]. This understanding of the sexual host’s function in generating novel pathogen variability became critical for cereal rust management strategies. Subsequent barberry eradication campaigns not only reduced disease incidence but also stabilized pathogen populations and delayed epidemic onsets [9]. However, complete eradication of the sexual host is not always feasible or environmentally desirable, especially in areas where they have significant ecological importance [10].

The ongoing challenge of managing cereal rusts highlights the significance of their complex biology, particularly their sexual reproduction cycle. This evolving knowledge of rust biology has shaped management strategies and emphasizes the need for continued research to address current and future challenges in cereal rust control.

## 2. When do cereal rusts have one or two nuclei?

**Fig 1:**
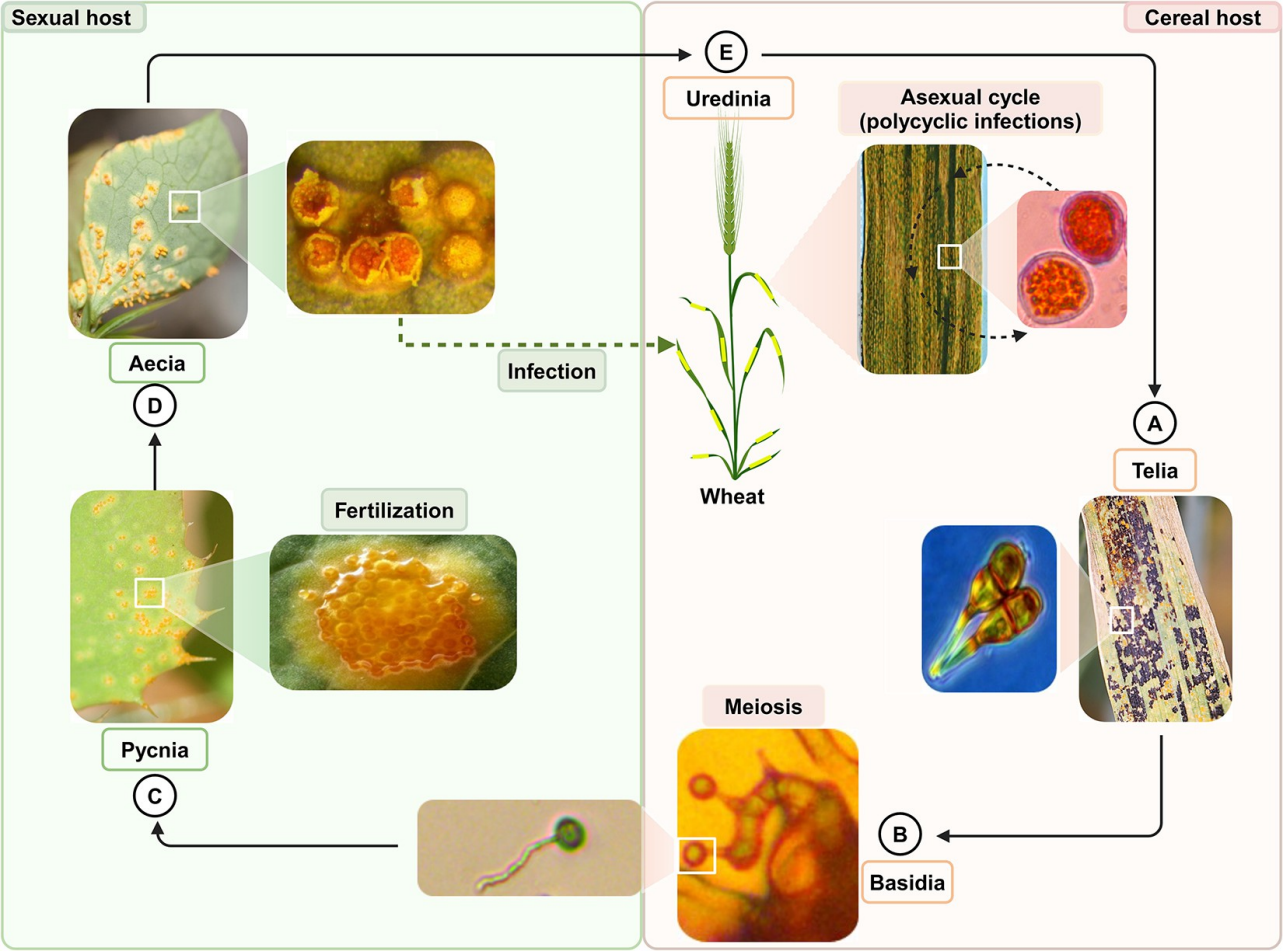
Schematic representation of the sexual and asexual life cycle of cereal rust fungi exemplified by the yellow rust fungus, *Puccinia striiformis* f. sp. *tritici*, infecting wheat and the sexual host, common barberry. (**A**) Telia containing two-celled teliospores are formed on the senescing wheat host. Initially, each cell contains 2 haploid (n) nuclei which then fuses to form a diploid (2n) nucleus in each cell after karyogamy. (**B**) Germinating teliospores bearing basidia with haploid (n) basidiospores of different mating types are formed after meiotic divisions. Germinated basidiospores may infect the sexual host. (**C**) Pycnia bearing haploid (n) pycniospores and receptive hyphae are formed on the adaxial side of the barberry leaf after basidiospore infection. Plasmogamy occurs when pycniospores fuse with receptive hyphae of different pycnia having different mating types. (**D**) Subsequently, aecia containing dikaryotic (n+n) aeciospores are formed on the abaxial side of the leaf which may infect the cereal host. (**E**) Uredinia containing dikaryotic (n+n) urediniospores are reproduced asexually in the cereal host, which may result in polycyclic infections within the same growing season (figure modified from [16,17]).

Cereal rust fungi exhibit a complex life cycle characterized by alternating nuclear states: haploid (n), dikaryotic (n+n), and diploid (2n) phases, occurring across both sexual and asexual life-stages on two botanically distinct host plants (Fig 1) [11].

The sexual life cycle is initiated with the formation of telia (specialized fungal structures producing thick-walled dikaryotic teliospores) on the senescing cereal host [1]. Within these structures, teliospores undergo nuclear fusion (karyogamy), becoming diploid. Teliospores then undergo meiosis, typically producing four basidiospores. In cereal rust fungi, basidiospores are generally both uninucleate and haploid (n). However, variations in nuclear number have been observed in some rust species [12]. Basidiospores infect the sexual host on the adaxial side of the leaves, leading to the formation of pycnia which contain haploid pycniospores and receptive hyphae. Plasmogamy (cell fusion) occurs when pycniospores fuse with receptive hyphae of opposing mating types (MAT) from different pycnia. This fusion leads to the formation of aecial clusters containing chains of dikaryotic aeciospores formed through mitotic divisions. The aeciospores then infect the cereal host, initiating the asexual phase of the life cycle. Subsequently, uredinia with dikaryotic urediniospores are formed. These urediniospores can reinfect the cereal host multiple times during a growing season, leading to rapid disease spread. As conditions become less favorable, the cycle completes with the formation of new telia on the cereal host [11].

The significance of these alternating nuclear states lies in the reproduction and evolutionary adaptability of cereal rust fungi. The haploid phase allows for the expression of individual alleles and generates diversity through mating type fusion. The dominant dikaryotic state maintains genetic variation by combining genetic material from two parental strains, with heterozygosity potentially influencing the expression of dominant and recessive alleles [13]. The transient diploid state facilitates multiple meiotic processes including genetic recombination that generates novel allele combinations, DNA repair, and removal of deleterious mutations through homologous recombination [14]. Population genetic studies have revealed patterns of pathogen diversity from sexual reproduction [15]. This diversity enables pathogens to respond to host resistance genes and environmental changes, shaping disease dynamics in agricultural systems. Understanding these nuclear dynamics is fundamental to unraveling how cereal rust pathogens adapt and persist in agricultural systems, which remains a significant challenge for disease management.

## 3. How to make two out of one?

In the phylum Basidiomycota, to which rust fungi belong, the transition from haploid to dikaryotic state is controlled by distinct mating-type genes. The MAT loci control mating-type and non-self recognition, which is essential for compatible gamete fertilization [18]. Two key loci are involved in mating-type determination in cereal rust fungi: the pheromone receptor (PR) locus and the homeodomain (HD) locus.

The PR locus contains genes encoding pheromone receptors (Pra) and mating pheromone peptide precursors (mfa) [18]. These genes are crucial for establishing pre-mating compatibility and facilitating gamete fusion. The HD locus consists of two closely linked genes encoding homeodomain transcription factors, typically referred to as bW-HD1 and bE-HD2, which regulate post-mating development, including the maintenance of the dikaryotic state [18].

The physical arrangement of the HD and PR loci varies among fungal species. In cereal rusts, recent research has revealed that the MAT loci are unlinked, supporting a tetrapolar mating system [19,20]. This finding challenges earlier assumptions of a bipolar system where the MAT loci would be physically linked [21]. The tetrapolar MAT system reduces mating compatibility compared to bipolar systems, as successful mating requires different alleles at both loci, resulting in only 25% sibling compatibility. While multiple MAT alleles can increase possible mating combinations, this allelic diversity is often limited in agricultural settings, potentially constraining sexual reproduction opportunities [19,22].

## 4. What do cereal rusts need to have sex?

Cereal rust fungi require specific environmental and biological conditions to complete their sexual reproduction cycle. First, teliospores, the entry point into the sexual life cycle, require particular conditions to break dormancy and undergo meiosis [12]. Dormancy periods vary among *Puccinia* species, influencing their ability to initiate the sexual cycle under different environmental conditions. For example, *Pgt* teliospores typically require a prolonged cold period to break dormancy, reflecting their adaptation to overwintering [11]. In contrast, *Pst* teliospores exhibit little to no dormancy, potentially germinating shortly after maturation [12].

Temperature and humidity requirements for teliospore germination and subsequent basidiospore production can vary significantly among rust species. For *Pgt*, teliospore germination occurs optimally between 15°C and 24°C, while *Pst* shows a lower optimal range of 10°C to 15°C [12]. *Puccinia coronata* teliospores show optimal germination at temperatures of 15 to 17°C. Humidity requirements for teliospore germination are generally high but species-specific. For instance, *Pgt* requires humidity levels typically above 98%, while *Pst* can tolerate slightly lower levels, around 95% [12,23]. Additionally, successful basidiospore infection of the sexual host requires prolonged periods of high humidity and leaf wetness across species, with the required duration varying among cereal rust species [24].

These high humidity requirements align with general basidiomycete biology, where moisture is essential for spore dispersal and germination [25]. High humidity periods typically coincide with the emergence of young leaves, synchronizing fungal sporulation with the presence of susceptible host tissue. Beyond environmental conditions, successful sexual reproduction requires synchronized susceptibility of both hosts, with basidiospore infection being most effective on newly expanded leaves of the sexual host [26]. This higher susceptibility of younger leaves influences the timing of infection and highlights the importance of synchronization between teliospore germination and the phenological stage of the sexual host. Spatial proximity between the cereal and the sexual hosts is also essential due to the limited dispersal capacity of basidiospores. Studies on *Melampsora lini*, a model pathosystem of rust fungi infecting wild flax, have demonstrated how spatial dynamics, including host distribution and pathogen dispersal patterns, influence infection success [27]. This spatial requirement highlights the importance of sexual host presence near cereal-growing regions for the completion of the sexual cycle [9]. The alignment of these factors, both temporally and spatially, determines the likelihood and frequency of sexual reproduction. The geographical distribution of sexual reproduction varies among species, with *Pgt* completing its sexual cycle across diverse regions while *Pst* is primarily limited to the Himalayan region [28]. Whether these geographical restrictions stem from specific environmental requirements, host adaptation, or other biological factors remains unclear, highlighting the complex interplay between pathogen biology and environmental conditions needed for successful sexual reproduction.

## 5. What remains unknown about sex in cereal rust fungi?

Despite significant advances in our understanding of cereal rust fungi, several crucial aspects of their sexual reproduction remain elusive. These knowledge gaps span from cellular and molecular processes to ecological implications.

At the cellular level, nuclear movement and fusion during sexual reproduction remains poorly characterized. Our understanding of how nuclei recognize each other and migrate within sexual structures, eventually leading to dikaryotization, is still limited. Research has revealed haploid and diploid nuclei coexisting within the same life cycle stage in *Puccinia* species, challenging traditional views of nuclear organization in rust fungi [29]. Advanced microscopy techniques targeting nuclear dynamics could help reveal these crucial cellular processes.

Major gaps persist in our understanding of molecular regulation of sexual reproduction in cereal rust fungi. The mechanisms controlling teliospore development, including how these fungi sense host senescence and initiate teliospore formation, remain poorly understood. Critical questions remain about dormancy regulation, including establishment, maintenance and how temperature changes trigger dormancy break. Additionally, the pathways regulating nuclear fusion during sexual reproduction and sexual structure formation require further investigation.

Understanding how sexual reproduction influences host adaptation in cereal rust pathogens remains challenging. Genetic and genomic studies are needed to identify how sexual recombination affects the inheritance of genes determining host range and how this process contributes to host specialization in cereal rust fungi. Perceived host specificity further raises questions about hybridization between closely related rust species that share sexual hosts, especially if they share compatible pheromone peptides and cognate receptors. Further, the dual-host life cycle introduces unique evolutionary challenges, particularly in maintaining distinct effector repertoires for two unrelated hosts. In asexual crop-infecting populations, genes required for sexual host infection may deteriorate without selection to maintain their function, potentially excluding these pathogen lineages from the sexual gene pool.

On a population level, the frequency and impact of sexual reproduction in different rust populations remain unclear. The extent of sexual reproduction in various geographic regions and its contribution to genetic diversity across rust species is not fully known. This knowledge gap affects our ability to predict the emergence of new virulent strains, with important implications for disease management and breeding strategies.

The environmental factors that trigger and regulate sexual reproduction need further study across cereal rust species. While temperature and humidity requirements are well characterized for *Pgt* and *Pst*, significant knowledge gaps exist for *Pt*, *Ph*, and *Pca*. The molecular and physiological mechanisms controlling teliospore dormancy, survival, and germination remain poorly understood in these species. Understanding how environmental cues and host phenology influence sexual reproduction success in agricultural landscapes is crucial, particularly under field conditions. Moreover, the potential impacts of climate change on these processes, including shifts in the timing and frequency of sexual events, require further study. The impact of habitat loss on rust genetic diversity remains poorly understood. A critical question is whether natural areas still serve as reservoirs of genetic diversity for recombination with rust populations adapted to modern agricultural systems, or whether these crop-associated pathogen populations represent the primary repository of genetic variation. Additionally, the role of somatic hybridization, where nuclear exchange occurs between pathogen strains without meiosis, needs further investigation. While documented in related rust fungi, the interaction between somatic hybridization and sexual reproduction in shaping population dynamics and pathogen adaptation remains to be fully characterized [30].

These knowledge gaps highlight the complexity of sexual reproduction in cereal rust fungi and key priorities for future research. Understanding these fundamental processes will advance our knowledge of rust biology, host adaptation, and pathogen evolution, and inform the development of more effective and durable disease management strategies.
